# Case report of whole genome sequencing in the XY female: identification of a novel *SRY* mutation and revision of a misdiagnosis of androgen insensitivity syndrome

**DOI:** 10.1186/s12902-016-0141-7

**Published:** 2016-11-08

**Authors:** Sunita M. C. De Sousa, Karin S. Kassahn, Liam C. McIntyre, Chan-Eng Chong, Hamish S. Scott, David J. Torpy

**Affiliations:** 1 Endocrine and Metabolic Unit, Royal Adelaide Hospital, Adelaide, SA Australia; 2 Department of Genetics and Molecular Pathology, Centre for Cancer Biology, an SA Pathology and UniSA alliance, Adelaide, SA Australia; 3 School of Medicine, University of Adelaide, Adelaide, SA Australia; 4 Hormones and Cancer Group, Garvan Institute of Medical Research, Sydney, NSW Australia; 5 School of Biological Sciences, University of Adelaide, Adelaide, SA Australia; 6 ACRF Cancer Genomics Facility, Centre for Cancer Biology, SA Pathology, Adelaide, SA Australia; 7 School of Pharmacy and Medical Sciences, University of South Australia, Adelaide, SA Australia

**Keywords:** Disorders of sexual development, Androgen insensitivity syndrome, Gonadal dysgenesis, Whole genome sequencing, Next generation sequencing, *SRY*, Case report

## Abstract

**Background:**

The 46,XY female is characterised by a male karyotype and female phenotype arising due to any interruption in the sexual development pathways *in utero*. The cause is usually genetic and various genes are implicated.

**Case presentation:**

Herein we describe a 46,XY woman who was first diagnosed with androgen insensitivity syndrome (testicular feminisation) at 18 years; however, this was later questioned due to the presence of intact Müllerian structures. The clinical phenotype suggested several susceptibility genes including *SRY*, *DHH*, *NR5A1*, *NR0B1*, *AR*, *AMH*, and *AMHR2*. To study candidate genes simultaneously, we performed whole genome sequencing. This revealed a novel and likely pathogenic missense variant (p.Arg130Pro, c.389G>C) in *SRY*, one of the major genes implicated in complete gonadal dysgenesis, hence securing this condition over androgen insensitivity syndrome as the cause of the patient’s disorder of sexual development.

**Conclusion:**

This case highlights the emerging clinical utility of whole genome sequencing as a tool in differentiating disorders of sexual development.

## Background

Disorders of sexual development (DSD) are categorised as 46,XY DSD, 46,XX DSD and chromosomal DSD (Table 1) with abandonment of pejorative terms such as *intersex* and *hermaphroditism*. DSDs may arise due to environmental factors such as maternal androgen exposure, or they may arise due to mutations in any of the genes involved in sexual development *in utero*. The leading nosology of the unvirilised 46,XY female are complete androgen insensitivity syndrome (CAIS) and complete gonadal dysgenesis (CGD). Whereas CAIS is due to mutations in the androgen receptor (*AR*) gene abolishing the actions of testosterone, CGD may be caused by various genetic aberrations leading to failure of testis development and thus testosterone production and aromatisation of testosterone to oestradiol. In both CAIS and CGD, effective testosterone deficiency results in female external genitalia, female body habitus and tall stature. Primary amenorrhoea is the commonest presentation in both conditions – due to Müllerian regression in CAIS and due to gonadal insufficiency in CGD [1]. Notably, milder forms of androgen insensitivity syndrome (AIS) and mixed or partial gonadal dysgenesis result in some degree of androgenisation, as do most androgen biosynthesis defects [1, 2].

**Table 1:** Overview of DSD categories including example conditions [1, 2]

DSD category	Examples	Karyotype or implicated genes
Chromosomal DSD	Klinefelter’s syndrome	47,XXY and variants/mosaics
	Turner’s syndrome	45,X and variants/mosaics
	Ovotesticular DSD	46,XX/46,XY chimerism
46,XY DSD	Gonadal dysgenesis	*DHH*, *SRY*, *NR5A1*, *NR0B1*, *WT1*, *SOX9*, *DMRT1*, *MAPK3K1*
	Androgen insensitivity syndrome	*AR*
	Disorders of androgen biosynthesis	*SRD5A2*, *STAR*, *CYP11A1*, *HSD3B2*, *CYP17A1*
	Structural genitourinary anomalies	*WNT4* (in Mayer-Rokitansky-Kuster-Hauser syndrome subset)
46,XX DSD	Disorders of ovarian development	*SRY, RSPO1, SOX9, WNT4*
	Disorders of androgen excess	*HSD3B2, CYP21A2, CYP11B1*

AIS is due to loss-of-function mutations in the *AR* gene positioned on the X chromosome and, with an incidence of up to 1/40,000 births, ﻿complete AIS (CAIS) is the commonest cause of the XY female. The molecular severity of the mutation dictates phenotypic severity, ranging from mild AIS with male phenotype and gynecomastia and/or infertility to CAIS with an unambiguously female external phenotype [3]. Androgen insensitivity *in utero* results in variable failure of testicular descent, which may manifest as bilateral inguinal herniae or vulval masses. Sertoli cell function is normal *in utero* and therefore anti-Müllerian hormone (AMH) production typically leads to Müllerian tract regression [1]; however, there are case reports of Müllerian remnants in CAIS suggesting a hitherto unrecognised role of the androgen receptor in AMH pathways [3]. Breast development should occur spontaneously in CAIS due to intact gonadal androgen production with aromatisation of testosterone to oestradiol [1].

CGD is characterised by streak gonads due to various genetic defects specific to testicular development (Table 1), including deletions and mutations in the sex-determining region of the Y chromosome (*SRY*) gene which account for 15 % of cases [1, 2]. The defect results in complete failure of testis development and a lack of gonadal hormone secretion from *in utero* onwards. Absence of AMH *in utero* results in Müllerian tract persistence and development [4]. Endogenous oestrogen deficiency prevents spontaneous breast development and may also cause the unstimulated uterus to be unidentifiable on imaging. Though androgen production ﻿is impaired in the gonads, it is intact in the adrena﻿ls, resulting in secondary sexual hair. Administration of oestrogen triggers breast development and uterine growth whilst cyclical oestrogen/progesterone induces withdrawal bleeds. CGD should be considered when an affected woman develops withdrawal vaginal bleeds with initiation of hormone replacement even if the baseline imaging failed to demonstrate a uterus; ultrasonography by radiologists or gynaecologists with special expertise in the area may be helpful to clarify any inconsistencies [1].

One of the major utilities of determining an accurate diagnosis in 46,XY DSD is in guiding gonadectomy since the risk of gonadal malignancy differs among XY females. As the tumour risk in CAIS is age-dependent and only amounts to 16 % over the long term in women with CAIS and intact gonads, there is an argument to consider gonadectomy postpubertally after spontaneous breast development through aromatisation of gonadal androgens and at an age where girls may be active participants in their healthcare [5]. In contrast, this is inappropriate in CGD where gonadal malignancy rates approximate 35 % with frequent occurrences in childhood [6]. Delay in gonadectomy is also inadvisable in partial AIS due to the risk of virilisation at puberty as there is only relative testosterone resistance [1].

The precise cause of 46,XY DSD may also influence decisions regarding hormone replacement as unopposed oestrogen therapy in CGD carries risks of endometrial hyperplasia and carcinoma due to stimulatory effects on the endometrium [7]. As hormone replacement in CGD is responsible for breast development and uterine growth, starting doses should be low and gradually increased to maximise the chances of normal breast contour and full uterine growth [4]. In contrast, oestrogen monotherapy may be used in CAIS as there is usually an absence of Müllerian structures, though this should be reviewed radiologically and at surgery given the aforementioned exceptions [3].

A diagnosis of CGD in 46,XY DSD may offer reproductive opportunities as successful pregnancies using donated ova have been achieved in women with CGD [4]. To date, this has not been described in CAIS where the Müllerian structures are rudimentary. One reproductive opportunity for women with CAIS in the future is use of their own gametes through in vitro fertilisation as testes in this condition have been found to contain spermatogonia, spermatocytes and spermatids [8]. This remains purely theoretical and ethically questionable given the risk of CAIS in offspring if gametes carrying the X chromosome and hence the *AR* mutation are used. Nonetheless, women with CAIS cited gamete preservation as a reason for declining gonadectomy in a survey of XY females, therefore this possibility may at least arise in patient-physician discussions [5].

There may be other clinical differences between CAIS and CGD which may only be unmasked by studying XY women with genetically-confirmed diagnoses as ambiguity in the clinical diagnosis is not uncommon. Of relevance to our patient who had type 2 diabetes mellitus, endogenous oestrogen deficiency in childhood and beyond appears to contribute to glucose intolerance. This might account for the disproportionately high rates of diabetes mellitus in gonadal dysgenesis and aromatase deficiency compared to the background population though how this compares to women with CAIS remains to be determined [9, 10].

The genetic evaluation of suspected DSD should begin with a karyotype. Fluorescence in situ hybridisation (FISH) may be performed simultaneously in the 46,XY female to confirm an intact *SRY* gene which is otherwise a straightforward cause of CGD. Subsequent investigations have traditionally involved single or staged gene analysis via Sanger sequencing guided by the clinical phenotype.

## Case presentation

An 18-year-old woman first presented for medical attention due to primary amenorrhoea. She was then a tall, thin woman with female external genitalia and no vulval or inguinal masses, however breast development and secondary sexual hair were conspicuously absent. The karyotype was 46,XY leading to a presumptive diagnosis of CAIS. Because of the suspicion of cryptorchid, dysgenetic gonads with high malignant potential, the patient underwent open laparotomy revealing streak gonads which were removed, and an infantile uterus which was left in situ. Histopathology demonstrated bilateral gonadoblastoma, and dysgerminoma in the right gonad. She received abdominopelvic radiotherapy with no tumour recurrence. After gonadectomy, she was commenced on the oral contraceptive pill and later changed to lower dose hormone replacement therapy.

At age 46 years, the patient migrated to Australia and sought review at our institution. Her active medical issues included type 2 diabetes mellitus, dyslipidemia, obstructive defecation syndrome and colonic polyps. She had a tall, slim build with a body-mass index of 20.4 kg/m^2^ and her proportions were eunuchoid with height 181 cm, arm span 182 cm and lower segment length 102 cm. There was minimal pubic hair and no axillary hair, but breast development was now normal. She had female external genitalia and a retroverted uterus.

**Fig. 1:**
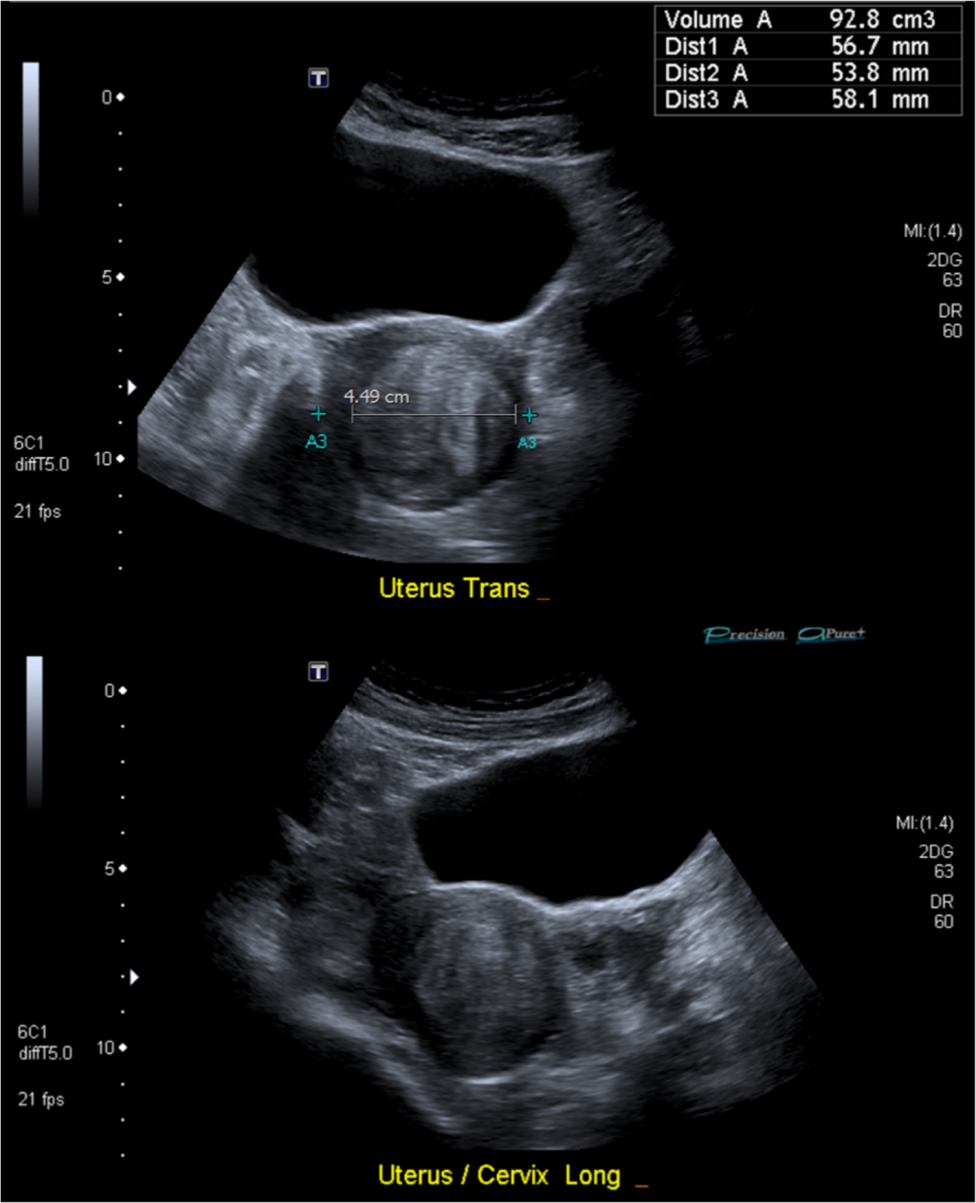
Transvaginal ultrasonography demonstrating a hypoplastic uterus with transverse (﻿to﻿p) and longitudinal (bottom) sections illustrating the globular shape. A 4.5 cm fibroid is seen within the uterine cavity, likely contributing to the patient’s menorrhagia

Results of hormonal studies and haematocrit were most consistent with hypogonadal female ranges (Table 2). Dual-energy x-ray absorptiometry demonstrated mild osteopenia with T-scores of -1.1 at the hip and spine. Because of menorrhagia, she underwent transvaginal ultrasound which demonstrated a fibroid within an hypoplastic uterus (Fig. 1). No renal tract anomalies were seen. After failed hysteroscopy and multiple alterations in hormone replacement, she was treated with transdermal oestradiol 25 mcg/hr and oral norethisterone 5 mg daily with cessation of menses at age 48 years.

**Table 2:** Results of biochemical tests

Test	Unit	Result	Female reference interval
βHCG	U/L	<2.0	<15.0^a^
AFP	kU/L	2	<7
Ca-19.9	kU/L	12	<37
AMH	pmol/L	<3	>14^b^
FSH	IU/L	53	>20^a^
LH	IU/L	24	>10^a^
Testosterone	nmol/L	1.1	<1.5^a^
SHBG	nmol/L	21	20–90^a^
Free androgen index	%	5.0	0.5–5.5^a^
Packed cell volume	L/L	0.39	0.35-0.45

^a^Postmenopausal range

^b^Premenopausal range only as postmenopausal status not applicable

It was now, in the patient’s fifth decade of life, that her original diagnosis of CAIS was questioned. Whilst the external female phenotype, XY karyotype and absence of secondary sexual hair were consistent with CAIS, the presence of streak gonads and functioning Müllerian structures along with the absence of spontaneous breast development favoured a diagnosis of CGD. Though Müllerian remnants have been observed in women with CAIS, there are no reports of the functioning uterine structures as found in our patient. We performed various genetic studies locally, beginning with a digital karyotype confirming the original 46,XY result. FISH demonstrated an intact *SRY* gene, excluding *SRY* deletion as the cause of the patient’s atypical DSD. Array comparative genomic hybridisation showed no copy number variation at an average effective resolution of 0.2 MB.

The major genetic possibilities at this stage were sequence variants and copy number variations in known CGD genes such as *DHH, SRY, NR5A1* and *NR0B1* [2]. In keeping with the patient’s original diagnosis overseas, synergistic mutations in *AR* and *AMH* or *AMHR2* were considered possible, as this would theoretically result in an unvirilised XY female phenotype with intact Müllerian structures. Whole exome sequencing was contemplated as the next step in our genetic evaluation, however this was deemed less suitable due to the non-contiguous capture and amplification steps which may hinder identification of copy number variations which are a significant cause of CGD [11]. Single nucleotide polymorphism (SNP) array was an alternative as its utility has been established in DSD [12], however the genetic resolution is low and does not allow for diagnosis of point mutations [11]. We instead opted for whole genome sequencing (WGS) in order to examine all susceptibility genes simultaneously and at high resolution. WGS was also favoured because of its capacity to detect copy number variations as the entire genome is contiguously analysed, allowing the detection of DNA breakpoints in cases of genetic deletions and duplications [11].

WGS was performed using the Illumina HiSeq X Ten Sequencing system at the Garvan Institute of Medical Research (Appendix). Bioinformatic analysis was performed at the SA Pathology Molecular Genetics Laboratory with variant classification by the American College of Medical Genetics 5-tier system: benign, likely benign, uncertain significance, likely pathogenic, or pathogenic [13].

Variants found on WGS were filtered based on mutational consequence (snpEff impact high or moderate) and phenotypic correlation. WGS revealed variants in eight genes associated with disorders of sexual development, namely: *AKR1C4*, *DMRT1*, *MAP2K1*, *MAP3K1*, *NR0B1*, *NR5A1*, *POR* and *SRY*. All but the *NR0B1* and *SRY* variants were excluded based on variant population frequencies >1 % in the 1000 Genomes (1 K), Exome Sequencing Project (ESP) and Exome Aggregation Consortium (ExAC) databases. The *NR0B1* variant was then excluded because of a population frequency >1 % amongst individuals in the ExAC database of the same ethnicity as the patient.

**Fig. 2:**
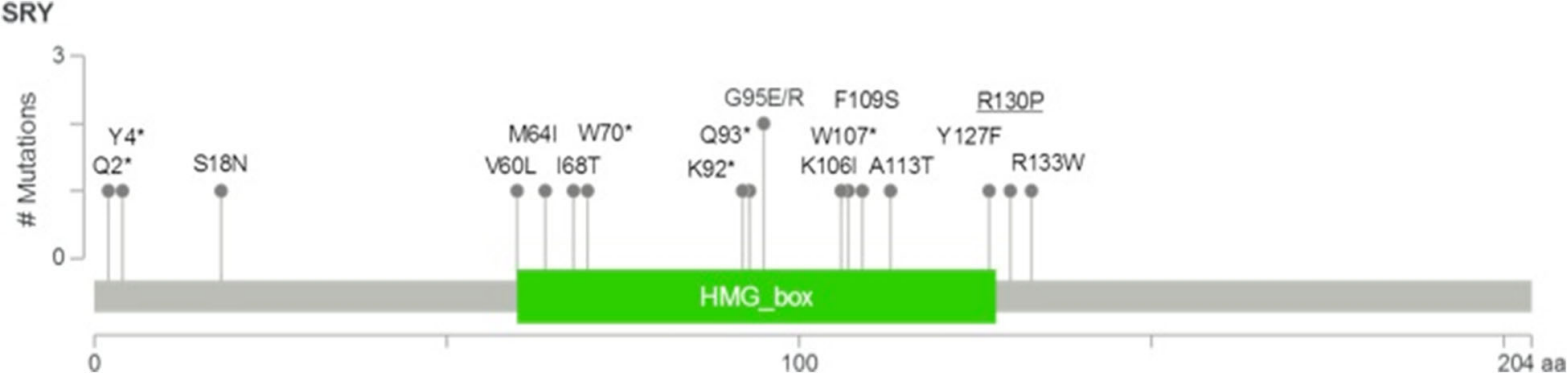
Domain graph of the novel *SRY* variant, R130P (underlined), found in the patient alongside pathogenic variants previously reported in ClinVar

**Fig. 3:**
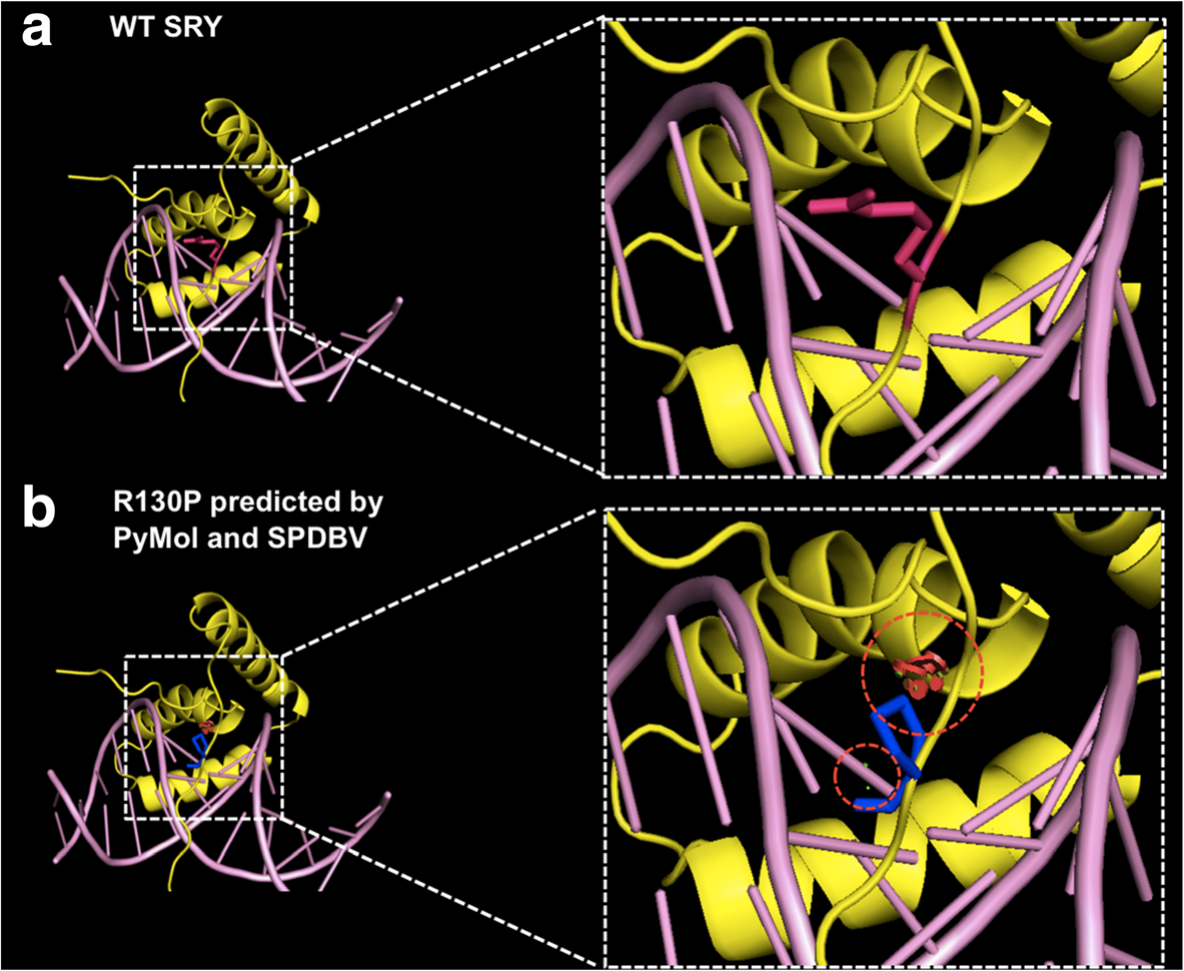
**a** Structure of human wild-type SRY protein in complex with the DNA. R130 (*pink*) is part of the random coil region of wild-type SRY protein. **b** The substitution R130P (*blue*) was shown through homology modelling to create steric hindrances (*red dotted lines*) to neighbouring residues

The remaining hemizygous missense variant in the *SRY* gene (NM_003140.2, p.Arg130Pro, c.389G > C) was confirmed to be present on Sanger sequencing of *SRY*. This variant has not been previously reported in the 1K, ESP, ExAC, or dbSNP databases, although there is an enrichment of pathogenic missense mutations at other locations of *SRY* reported in the ClinVar database (Fig. 2). It is located at the 3’ end of the HMG box domain in a region required for nuclear localisation (UniProt Q05066) and it is predicted to be pathogenic by PolyPhen2 and MutationTaster, but not SIFT. It is moderately conserved (phyloP 3.43, CADD 10.44) and has a moderate Grantham difference of 103. Protein-DNA complex modelling demonstrated that substitution of arginine (Arg) to proline (Pro) causes steric hindrances to the native SRY protein structure which may affect DNA binding (Fig. 3a and b).

The evidence collectively demonstrated this novel *SRY* variant to be likely pathogenic, which is consistent with a diagnosis of 46,XY DSD (OMIM 400044), and more specifically CGD, thus revising the patient’s diagnosis from CAIS to CGD 30 years after her original presentation. The patient’s Müllerian structures were now considered a consistent feature of her DSD, although the reason for the absence of secondary sexual hair remains unclear as we excluded a concomitant mutation in the *AR* gene on WGS.

## Conclusions

In the presented case, the patient was phenotypically female with no evidence of virilisation and we confirmed a 46,XY karyotype. The leading differentials were therefore CAIS and CGD. Whilst the absence of secondary sexual hair was consistent with CAIS, the presence of streak gonads and functioning Müllerian structures along with the absence of spontaneous breast development were more suggestive of CGD contrary to her original diagnosis. To overcome the issues of multiple susceptibility genes and the possibility of either point mutations or copy number variations, we performed WGS which identified a novel *SRY* mutation thus securing the diagnosis of CGD over CAIS. If we were to have performed single gene analysis based on the patient’s original diagnosis, the *AR* gene would have been sequenced with no yield. In current clinical practice, the cost of WGS is approximately US$2600 with the exact price depending on laboratory throughput and the technologies employed. The cost is generally less in research settings and further decreases are expected with improving efficiency in the sequencing and bioinformatic processes. With the increasing availability, cost-effectiveness, speed and understanding of WGS, we support its use in the clinical evaluation of the XY female due to the presence of genetic heterogeneity and oftentimes atypical clinical features in the DSD spectrum.

## Appendix

### Genetic sequencing methodology

Germline DNA was extracted from blood using a QiaAMP DNA Mini kit (Qiagen) at SA Pathology, Adelaide, Australia. WGS was performed by the Garvan Institute of Medical Research, Sydney, Australia using the Illumina HiSeq X Ten Sequencing system and paired-end 2x150bp sequencing. A total of 827,574,432 and 835,216,904 reads equating to 62.48 Gb and 63.06 Gb of raw sequence, respectively, were generated. Bioinformatic analysis was performed at the SA Pathology Molecular Genetics Laboratory. Reads were mapped to the human genome (build hg19) using Burrows-Wheeler Aligner (BWA) Version 0.7.9a, duplicates marked, indels realigned and quality scores recalibrated using Picard (V1.71).

Variants were called in exonic regions using the HaplotypeCaller in the Genome Analysis Toolkit (GATK) Version 3.3.0 following the GATK Best Practice Guidelines. A mean coverage of 51.89x was achieved in RefSeq exons. Functional annotation was carried out using snpEff and snpSift (Version 3_3) and Ensembl GRCh37.70 annotation. Variants were further annotated with information from the 1000 Genomes Project (integrated phase 1 data, Version 3), Exome Variant Server (ESP6500SI-V2), dbSNP (build 137), OMIM and GO terms. Variants were filtered based on minor allele frequency (requiring less than 1 % in the global individuals from the 1000 Genomes Project) and mutational consequence (snpEff impact high or moderate).

After discarding variants with unrelated phenotype, eight resulting variants were manually assessed for disease relationship by examining the OMIM terms and ExAC allele frequency (http://exac.broadinstitute.org/) and each was ranked in order of likelihood of being disease-related. Variant classification by the American College of Medical Genetics 5-tier system incorporated data from conservation models (Grantham, CADD, phyloP), *in silico* pathogenicity prediction (PolyPhen, SIFT, AlignGVGD, MutationTaster, Alamut), biophysicochemical properties (UniProt domain annotations, structural models), published literature, and mutation databases (ClinVar, HGMD). *In silico* mutagenesis of protein-DNA complex (pdb: 1j46.1) was performed using PyMol and spdbv version 4.10 software.

Validation of the final WGS result was performed via bidirectional Sanger sequencing of the *SRY* gene at Flinders Medical Centre, Adelaide, Australia.

## Abbreviations

AIS: Androgen insensitivity syndrome
AMH: Anti-Müllerian hormone
AR: Androgen receptor
CAIS: Complete androgen insensitivity syndrome
CGD: Complete gonadal dysgenesis
DSD: Disorder of sexual development
FISH: Fluorescent *in situ* hybridization
SNP: Single nucleotide polymorphism
SRY: Sex-determining region of the Y chromosome
WGS: Whole genome sequencing
